# Light-activated Multilevel Resistive Switching Storage in Pt/Cs_2_AgBiBr_6_/ITO/Glass Devices

**DOI:** 10.1186/s11671-021-03636-6

**Published:** 2021-12-13

**Authors:** Tingting Zhong, Yongfu Qin, Fengzhen Lv, Haijun Qin, Xuedong Tian

**Affiliations:** School of Physical Science and Technology and Guangxi Key Laboratory of Nuclear Physics and Technology, Guangxi Normal University, Yucai Road, Guilin, 541000 China

**Keywords:** Light regulation, Cs_2_ AgBiBr_6_, Multilevel resistive switching, Bromine vacancy, Space charge-limited current mechanism, Schottky-like barrier

## Abstract

**Abstract:**

High-density Cs_2_AgBiBr_6_ films with uniform grains were prepared by a simple one-step and low-temperature sol–gel method on indium tin oxide (ITO) substrates. An explicit tristate bipolar resistance switching behavior was observed in the Pt/Cs_2_ AgBiBr_6_/ITO/glass devices under irradiation of 10 mW/cm^2^ (445 nm). This behavior was stable over 1200 s. The maximum ratio of the high and low resistance states was about 500. Based on the analysis of electric properties, valence variation and absorption spectra, the resistive switching characteristics were attributed to the trap-controlled space charge-limited current mechanism due to the bromine vacancies in the Cs_2_AgBiBr_6_ layer. On the other hand, it is suggested that the ordering of the Schottky-like barrier located at Pt/Cs_2_AgBiBr_6_ affects the three-state resistance switching behavior under light irradiation. The ability to adjust the photoelectrical properties of Cs_2_AgBiBr_6_-based resistive switching memory devices is a promising strategy to develop high-density memory.

**Graphical Abstract:**

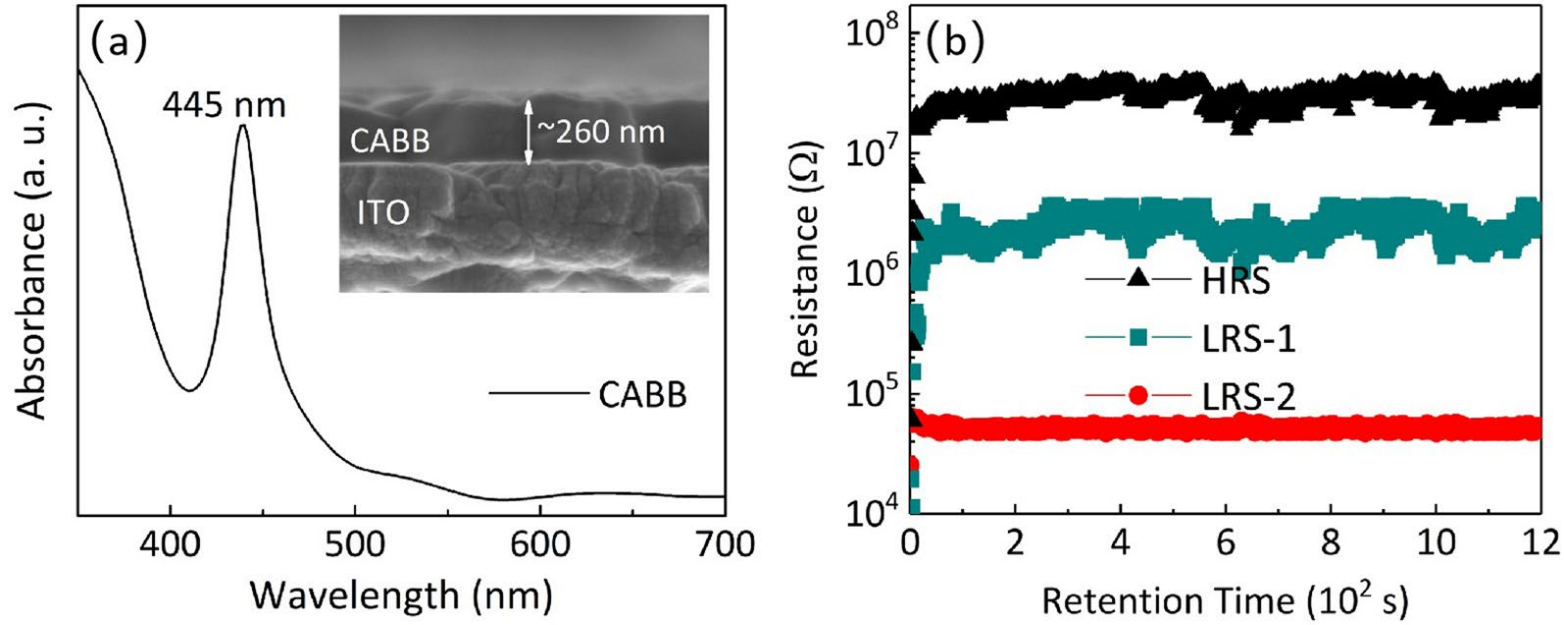

**Supplementary Information:**

The online version contains supplementary material available at 10.1186/s11671-021-03636-6.

## Introduction

With the increase of redundancy and complexity of information, demands for higher density, faster speed, smaller scale and lower consumption have brought about new development of new concept of information storage [1, 2]. The nonvolatile resistive random access memory (RRAM) is the most promising data storage candidate due to its simple structure, high write/read speed and low-scale limit and power consumption [2–4]. RRAM devices operated by a single electrical input have been widely researched and reported. However, this single input method faces the problem of the discovery of the appropriate media layer, the lowering of the power consumption and the problem of the multifunction expansion [5, 6]. Thus, the exploration of storage strategies that combine multiple physical channels, *e*.*g*., optical, electric and magnetic fields, has been extensively undertaken [5–8]. Among them, the optical field is used as an extra terminal of a photoelectric materials-based RRAM device to ensure large memory windows and multiple storage levels greatly [5, 6, 9, 10]. Among various photoelectric materials, lead-based perovskites ($\hbox {APbX}_{{3}}$; with A =$\hbox {Cs}^{+}$, $\hbox {CH}_{{3}} \hbox {NH}_{3}^{+}$, ($\hbox {H}_{{2}}$N)$_{2} \hbox {CH}^{+}$, *etc*.) have been widely researched in photonic memristors due to their fast optical response, high photoelectric conversion efficiency and large defect tolerance [11–14]. However, the toxicity of soluble lead content and the long-term instability limit the broad application of $\hbox {APbX}_{{3}}$-based photoelectronic memories [15, 16]. Above hindrances have triggered the exploration of lead-free alternatives with higher stability. Recently, lead-free double perovskites have been proposed in the chemical formula $\hbox {A}_{{2}} \hbox {M}^{+} \hbox {M}^{3+} \hbox {X}_{{6}}$, where $\hbox {M}^{+}$ (monovalent cation) and $\hbox {M}^{3+}$ (trivalent cation) are used to replace $\hbox {Pb}^{2+}$ [17, 18]. Here, silver-bismuth double perovskites (e.g., $\hbox {Cs}_{{2}} \hbox {AgBiBr}_{{6}}$ and $\hbox {Cs}_{{2}} \hbox {AgBiCl}_{{6}}$) have extensively aroused concern due to the higher chemical stability, lower toxicity and more excellent optoelectronic property [15, 19, 20]. Although the potential for solar cells, photovoltaic devices and photoelectric detectors has been studied, photoelectric storage applications are not fully utilized [21–23].

In this study, high-density $\hbox {Cs}_{{2}} \hbox {AgBiBr}_{{6}}$ (CABB) films with high coverage were prepared by a simple one-step and low-temperature sol–gel method on indium tin oxide (ITO) substrates. Tristate RS characteristics, one high resistance state (HRS, i.e., OFF state) and two low resistance states (LRSs, i.e., ON states), were achieved in the Pt/CABB/ITO/glass devices under the light illumination (445 nm) of 10 mW/$\hbox {cm}^{2}$. The maximum resistance ratio between HRS and LRSs reached about 500. The current behavior was controlled by trap-controlled space charge-limited current (SCLC) mechanism owing to abundant bromine vacancies (V$_\mathrm{Brs}$) in the CABB layer. Under light illumination, bias voltage sweep caused more V$_\mathrm{Brs}$ to be formed and captured/released electrons during the trapping/detrapping processes. In addition, the change of Pt/CABB Schottky-like barrier by the optical modulation also contributes to the multilevel RS behavior. This work demonstrates that CABB has the potential application in light-assisted multilevel storage devices. Related findings also provide an opportunity to understand the inherent nature of lead-free halide perovskites for their usage in high-density and nonvolatile optoelectronic memories.

## Methods

**Fig. 1:**
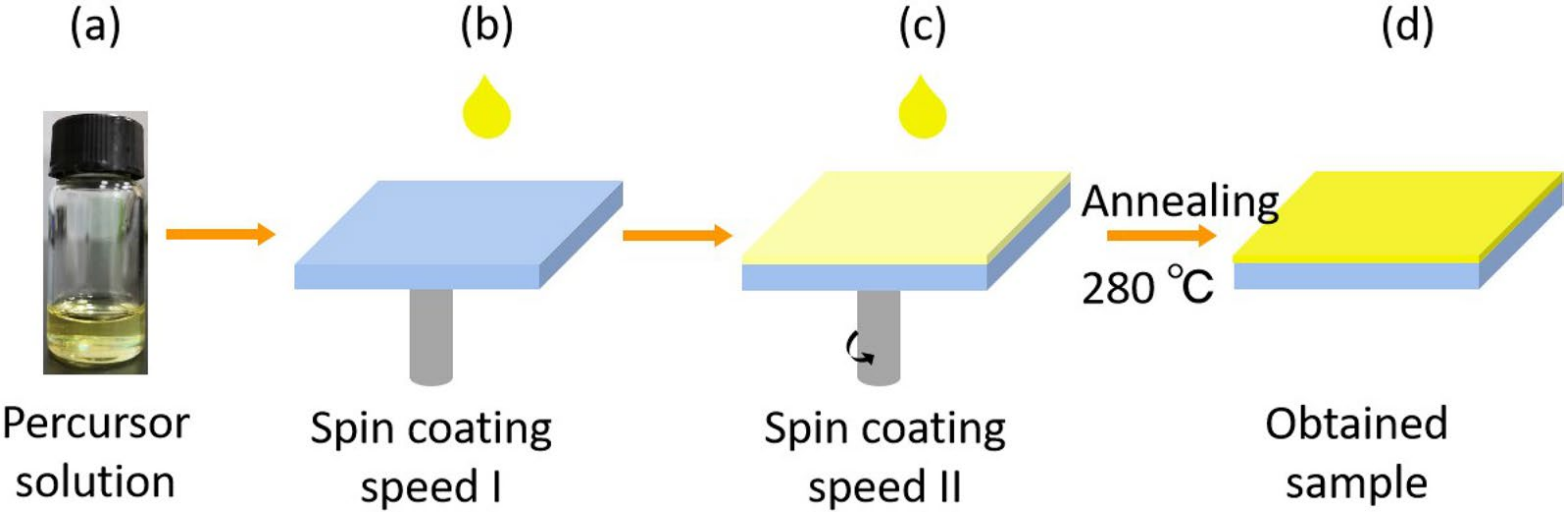
Solvent engineering procedure for preparing the CABB/ITO/glass sample

Prior to the growth of the samples, the ITO/glass substrate was washed sequentially with acetone, isopropyl alcohol and deionized water and dried under a nitrogen gas flow. In the anhydrous dimethyl sulfoxide (DMSO, 1mL), 1.2 mmol of CsBr, 0.6 mmol of AgBr and 0.6 mmol of $\hbox {BiBr}_{{3}}$ were dissolved. The yellow precursor solution was obtained after stirring for 12 h at 65 $^{\circ }$C in an Ar-filled glove box, as shown in Fig. 1a. The precursor solution was dripped onto an ITO/glass substrate and spin-coated at 500 rpm (speed I) for 15 sec and 5000 rpm (speed II) for 45 sec [Fig. 1b, c]. Finally, as shown in Fig. 1d, the yellowish film was obtained by annealing on a hot plate at 280 $^{\circ }$C for 5 min.

## Characterization

The crystallinity and phase purity of samples were investigated using X-ray diffraction (XRD, MiniFlex600, Rigaku Corporation, Japan) with a Cu K$\alpha$ radiation at room temperature. The compositional analysis and band structure of Pt/CABB/ITO cells were performed by X-ray photoelectron spectroscopy (XPS) and Ultraviolet photoelectron spectroscopy (UPS) with a monochromatic Al $\hbox {K}_{\alpha }$ X-ray source, respectively (XPS/UPS, ESCALAB250Xi, Thermo Fisher Scientific, America). A UV–Vis reflectance spectrum was recorded using a UV-2700 spectrophotometer (Shimadzu Corporation, Japan). Microscope images of the CABB film morphologies were recorded by a field-emission scanning electron microscope (SEM, FEI Quanta 200, FEI, Holland). All current–voltage (*I*–*V*) characteristics of the cells in Pt/CABB/ITO/glass configuration were examined using a Keithley 2400 SourceMeter.

## Results and Discussion

**Fig. 2:**
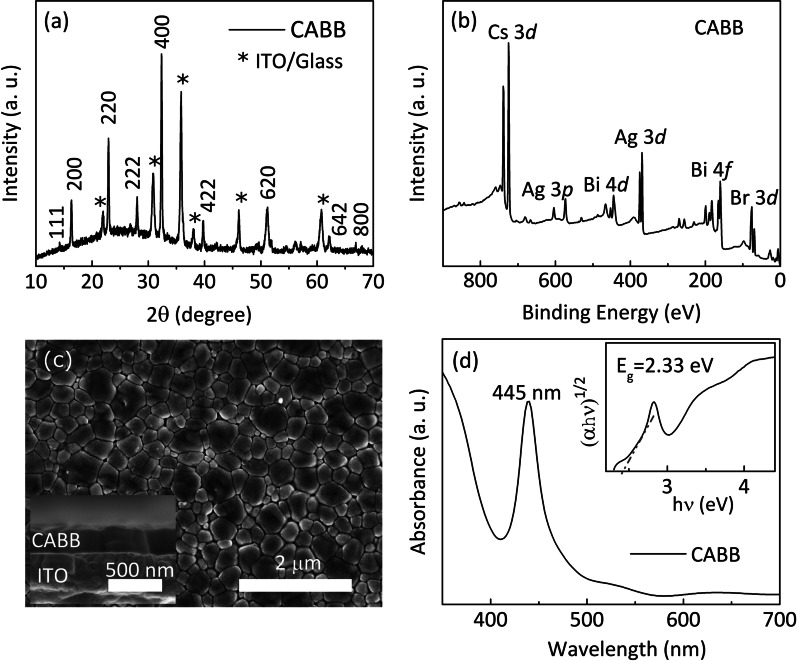
**a** The longitudinal $\theta-2\theta$ scans of the as-grown CABB film on the ITO/Glass substrate. $*$ represents peaks of the ITO/glass substrate. **b** XPS spectrum of CABB. **c** The top-view SEM image of films. The inset displays the cross-sectional SEM image of the CABB film. **d** Optical absorption spectrum of the CABB thin film measured at room temperature

Figure 2a exhibits that the XRD peaks are distributed in the (111), (200), (220), (222), (400), (422), (620) and (642) planes of crystalline CABB, corresponding to the cubic phase of CABB [24–26]. The wide spectrum of XPS clearly presents only Cs, Ag, Bi and Br elements in the samples, indicating that the chemical composition of the CABB film is pure [Fig. 2b]. The top view of the surface morphology reveals that CABB films possess a high surface coverage and relatively uniform crystal grains. Many obvious grain boundaries provide channels to the migration of ions and vacancies. And these grain boundaries can promote a further reduction in conductive energy barriers of ions and vacancies due to their light sensitivity [27, 28]. The CABB layer which has a uniform thickness of $\sim$260 nm is clearly visible in the inset of Fig. 2c. UV–Vis absorption spectrum indicates that an excitonic absorption band in the region from 420 to 500 nm, the peak locates at 445 nm [Fig. 2d].

**Fig. 3:**
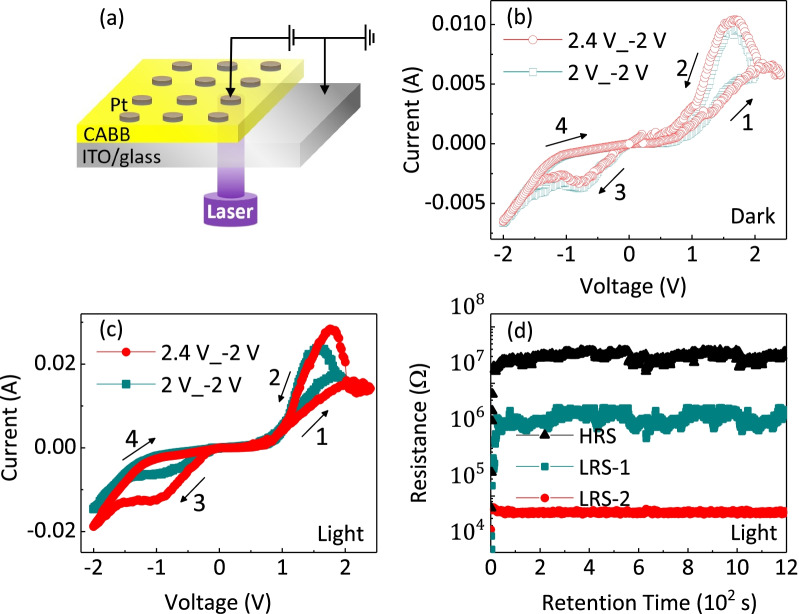
**a** The configuration of the Pt/CABB/ITO/glass memory device. I–V curves for the device in dark (**b**) and under light irradiation (**c**), respectively. The numbers and arrows represent the voltage sweeping sequences and directions, respectively. **d** Time up to over 1200 s for HRS, LRS-1 and LRS-2 measurements under the light illumination of 445 nm at room temperature

Figure 3a displays the schematic structure of the fabricated CABB-based device under violet light (445 nm) of 10 mW/$\hbox {cm}^{2}$, where a CABB layer is sandwiched between the Pt-based top electrode (TE) and the ITO-based bottom electrode (BE). The bias voltage is applied on the Pt TE of the device, while the ITO BE is grounded. As exhibited in Fig. 3b, by applying voltage loops to the Pt/CABB/ITO/glass device with a periodic sweeping (0 V$\rightarrow$2 V/2.4 V$\rightarrow$-2 V$\rightarrow$0 V), an obvious current hysteresis is observed during the voltage sweeping in the dark condition. The device presents a typical bipolar RS behavior. However, different resistance states are not observed under different operating voltages. When the device was exposed in the light with a laser of 445 nm, 10 mW/$\hbox {cm}^{2}$, the current distinctly switched between two correspondingly different LRSs in the negative region, as shown in Fig. 3c. The device presents obvious tristate RS behavior under the light illumination. Three different resistance states, HRS, LRS-1 and LRS-2, were recorded at a low reading voltage ($V_\mathrm{r}=-0.1$ V) under light illumination. As illustrated in Fig. 3d, the maximum resistance ratio of HRS and LRSs is about 500 at $V_\mathrm{r}$. HRS, LRS-1 and LRS-2 can be stably maintained and no obvious degradation occurs after 1200 s. The above results illustrate that Pt/CABB/ITO/glass devices possess the fine retention ability and the multilevel storage potential. In order to confirm the optimal condition of light-activated multilevel RS characteristics, we measured the RS behavior of Pt/CABB/ITO/glass devices under different light intensities (Additional file 1: Fig. S1). Compared with the *I*–*V* curve measured under the light intensity of 10 mW/$\hbox {cm}^{2}$ [Fig. 3d], different LRSs could not be obviously observed in the negative bias voltage region under the light intensities of 8 mW/$\hbox {cm}^{2}$ and 13 mW/$\hbox {cm}^{2}$, as shown in Additional file 1: Fig. S1a, b. Moreover, as the light intensity increases, although the multilevel RS characteristics appear, the maximum ratio of HRS and LRSs under the intensities of 8 mW/$\hbox {cm}^{2}$ is 13 mW/$\hbox {cm}^{2}$ are just $\sim$10 and $\sim$3, respectively [Additional file 1: Fig. S1c, d]. And the stability of multilevel RS characteristics under these intensities is not good as that of 10 mW/$\hbox {cm}^{2}$. Aiming at the influence and law of different light intensities for the RS behavior of CABB-based memories will be researched and discussed in our next work.

**Fig. 4:**
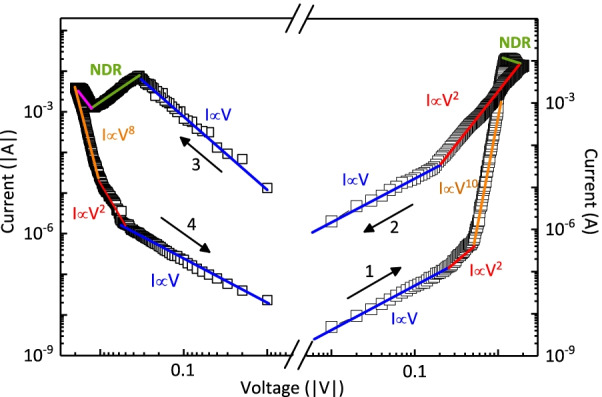
The fitted lines of the double logarithmic *I*–*V* plots. The arrows indicate sweeping directions

The bipolar RS behavior in different memories can be normally explained by three types of mechanisms, namely the electrochemical metallization mechanism (ECM), the valence variation mechanism (VCM) and the thermochemical mechanism (TCM) [12, 29]. In the ECM model, the RS switching behavior is based on the formation or rupture of metallic conductive filaments formed by active metal atoms within the medium layer [12, 30]. However, the inert Pt metal was applied to be as the electrode of the CABB-based memory device in our work; thus, we speculated that the VCM model was primarily contributed to the RS behavior in the Pt/CABB/ITO/glass device. As illustrated in Fig. 4, the *I*–*V* curve is replotted on double logarithmic coordinates in the positive and negative voltage sweep regions. In the positive region, the fitting slope of log(*I*)–log(*V*) curve is approximately 1 in the voltage range of 0 and 0.3 V, the *I*–*V* relationship obeys the Ohm’s law. With the positive voltage increasing, the slopes of the fitted lines are approximately 2 and 10 in the voltage range of 0.3 V and 1.1 V, respectively, indicating that the SCLC model dominates the conductive process [12, 30, 31]. With the positive bias further increasing in the range of 1.1 V and 1.9 V, the current decreases obviously, indicating the so-called negative differential resistance (NDR) appearing [32]. When the positive bias reaches $V_\mathrm{SET}$ (+2 V), the device switches from HRS to LRS, and then, the relationship of log(*I*)–log(*V*) obeys ${I}\propto {V}^{2}$ (1.8 V–0.2 V). Subsequently, a linear relation is quickly observed between *I* and *V*. The conductive behavior obeys Ohmic conduction even though the bias reversely sweeps. When the device switches from LRS to HRS under $\textit{V}_\mathrm{RESET}$ (−2 V), the slope of the fitted curve recovers about 8 and 2 in the range of −2 V and −0.5 V, respectively, suggesting that the SCLC mechanism dominates this HRS region. With the negative voltage decreasing further, the *I*–*V* follows the Ohmic conduction.

**Fig. 5:**
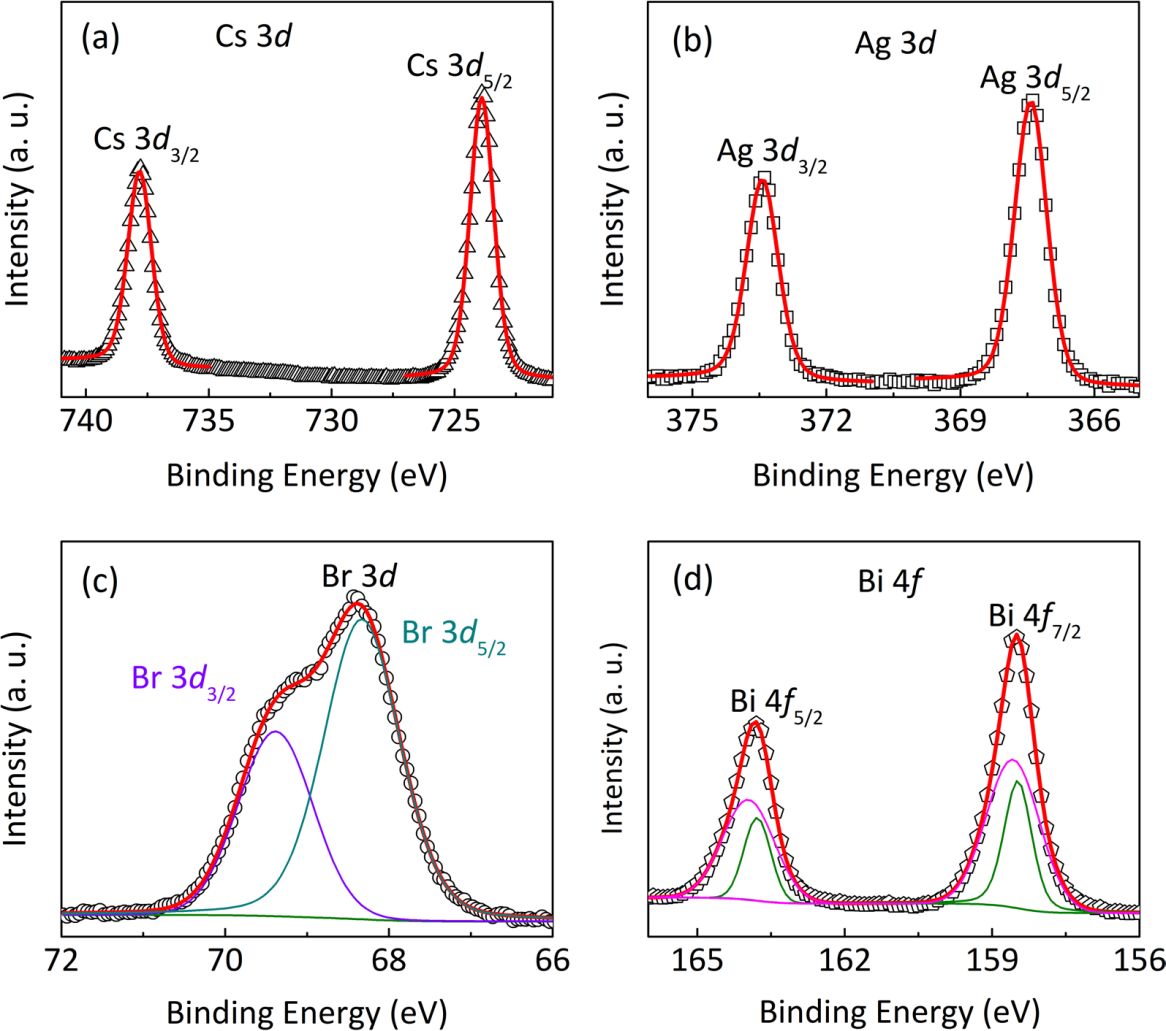
XPS data corresponding to **a** Cs 3$\textit{d}$, **b **Ag 3$\textit{d}$, **c** Br 3$\textit{d}$ and **d **Bi 4$\textit{f}$ of the CABB films

To trace and confirm the origin of SCLC conductive mechanism in the Pt/CABB/I TO/ glass device, XPS measurement was taken to investigate the valence variation of the fabricated CABB films. As shown in Fig. 5a, two clear peaks at 737.83 eV and 723.88 eV are observed corresponding to $\hbox {Cs}^{+}$ [33]. The presence of $\hbox {Ag}^{+}$ is also revealed according to the peaks located at 373.43 eV and 367.42 eV ascribed to 3$\textit{d}_{3/2}$ and 3$\textit{d}_{5/2}$, respectively [Fig. 5b]. The fitting results of Cs and Ag elements are consistent with the valence composition in the CABB films. As depicted in Fig. 5c, the doublet peaks of Br 3$\textit{d}_{3/2}$ and Br 3$\textit{d}_{5/2}$ signals locate at 69.38 eV and 68.33 eV, respectively. Obviously, they shift toward the positive positions, indicating that V$_\mathrm{Brs}$ generate in the CABB layer [34]. In addition, as illustrated in Fig. 5d. Bi 4$\textit{f}_{5/2}$ and Bi 4$\textit{f}_{7/2}$ regions are fitted into two peaks, respectively. The main peaks at 163.96 eV and 158.60 eV are assigned to $\hbox {Bi}^{3+}$, and two peaks with lower binding energy at 163.78 eV and 158.48 eV are ascribed to the low valence state $\hbox {Bi}^{(3-x)+}$, indicating the generation of V$_\mathrm{Brs}$ in the CABB layer [35].

**Fig. 6:**
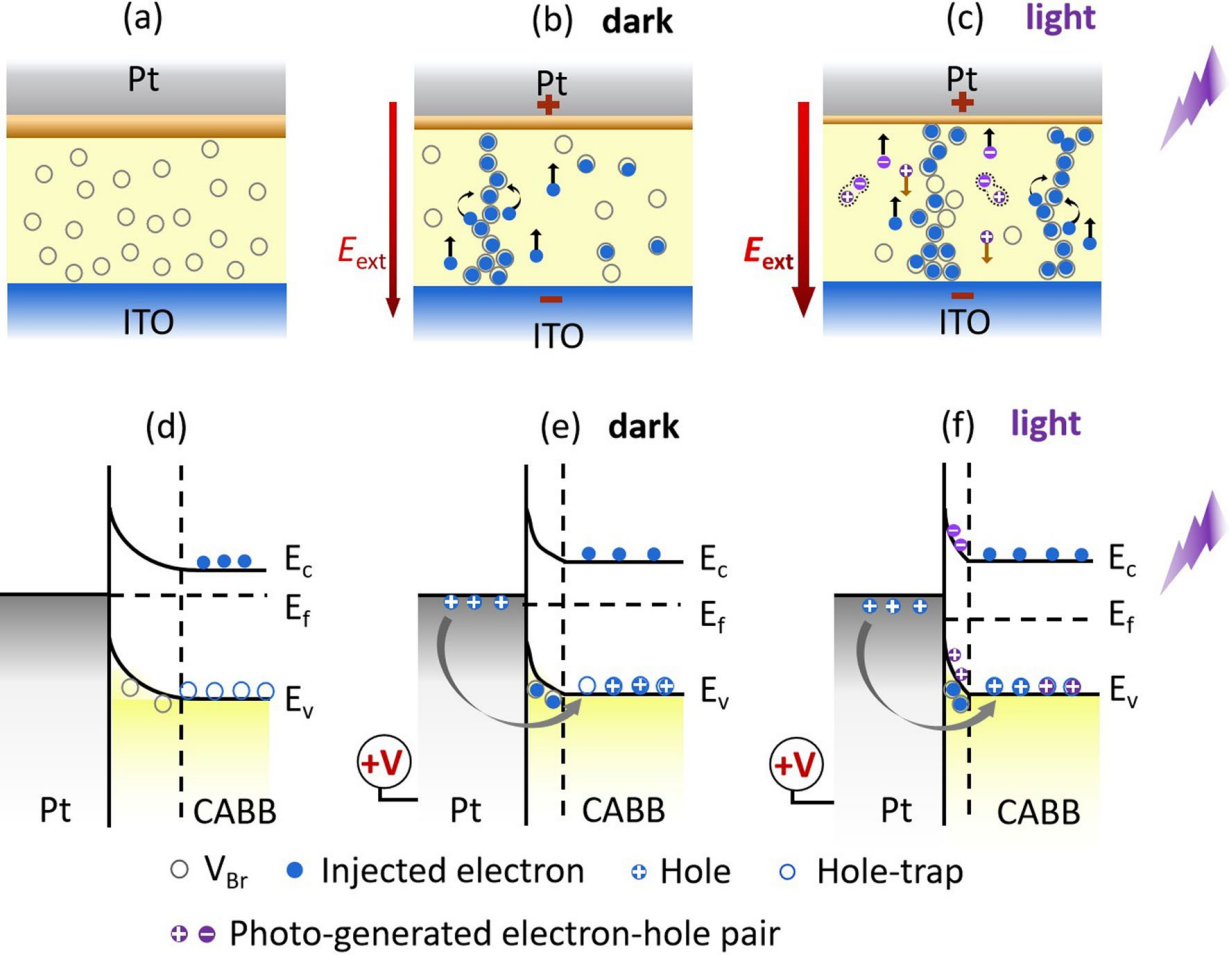
The electric conductive process and the schematic band diagram of the Pt/CABB/ITO/glass device in the (**a**) and **b** absence, **c** presence of light

Initially, the Pt/CABB/ITO/glass device is difficult to conduct electricity and stays in the HRS due to V$_\mathrm{Brs}$ randomly disperse in the CABB layer [Fig. 6a]. When a low positive bias is applied to the Pt TE, an external electric field ($E_\mathrm{ext}$) is formed in the CABB layer, which points from Pt TE to ITO BE. The *I*–*V* relationship follows Ohm’s law own to the conduction of thermally excited electrons in the CABB layer. With the $E_\mathrm{ext}$ increasing, the electrons injected from the ITO BE are captured by V$_\mathrm{Brs}$ in the CABB layer, the relationship of *I*–*V* follows Child’s low (${I}\propto {V}^{2}$), as shown in Fig. 6b. With $E_\mathrm{ext}$ increasing further, the $\hbox {Br}^{-}$ ions are attracted to the Pt TE, creating more V$_\mathrm{Brs}$ to capture electrons during the trapping process. When the positive bias arrives $V_\mathrm{SET}$, V$_\mathrm{Brs}$ form conduction channels due to the lowest activation energy; the injected electrons can migrate by vacancy to vacancy hopping [9, 36]. The device switches from HRS to LRS and remains Ohmic conduction even though the bias sweeps reversely. While the Pt/CABB/ITO device is exposed under the light irradiation of 445 nm, photoinduced electron–hole pairs generate in the CABB layer [Fig. 6c]. With the $E_\mathrm{ext}$ increasing, electron–hole pairs are divided. Photo-generated holes can recombine with $\hbox {Br}^{-}$, promoting the generation of V$_\mathrm{Brs}$ in the CABB film [37]. In the meantime, the driving force (*zeE*) that drives the migration of V$_\mathrm{Brs}$ is enhanced, causing the activation energy of V$_\mathrm{Brs}$ lowering by [$-\varepsilon$/2 ($\varepsilon \propto zeE$)]; more V$_\mathrm{Brs}$ generate in the CABB film [27, 38]. Thus, more conductive paths composed of V$_\mathrm{Brs}$ form and participate in the RS process under bias voltage sweeping. When an opposite voltage is applied to the Pt TE and goes across $V_\mathrm{RESET}$, the trapped electrons are drawn out from V$_\mathrm{Brs}$, and the current behavior obeys the SCL conduction. The relationship of *I*–*V* follows $\textit{I}\propto \textit{V}^{2}$. Meanwhile, the bridges of V$_\mathrm{Brs}$ are ruptured resulting from the reverse migration of $\hbox {Br}^{-}$ ions influenced by the negative voltage. And thus, the device switches from LRS to HRS. The above evolution of $\hbox {Br}^{-}$ conductive channels has been measured and researched by the conducting atomic force microscopy in our previous work [26]: When a positive bias voltage was applied to the CABB film, obvious current channels were observed, indicating that the conductive filaments were formed in the CABB layer; when a negative bias voltage was applied to scan the same area, the layer could not present obvious current, suggesting that the conductive filaments were ruptured under the negative voltage.

**Fig. 7:**
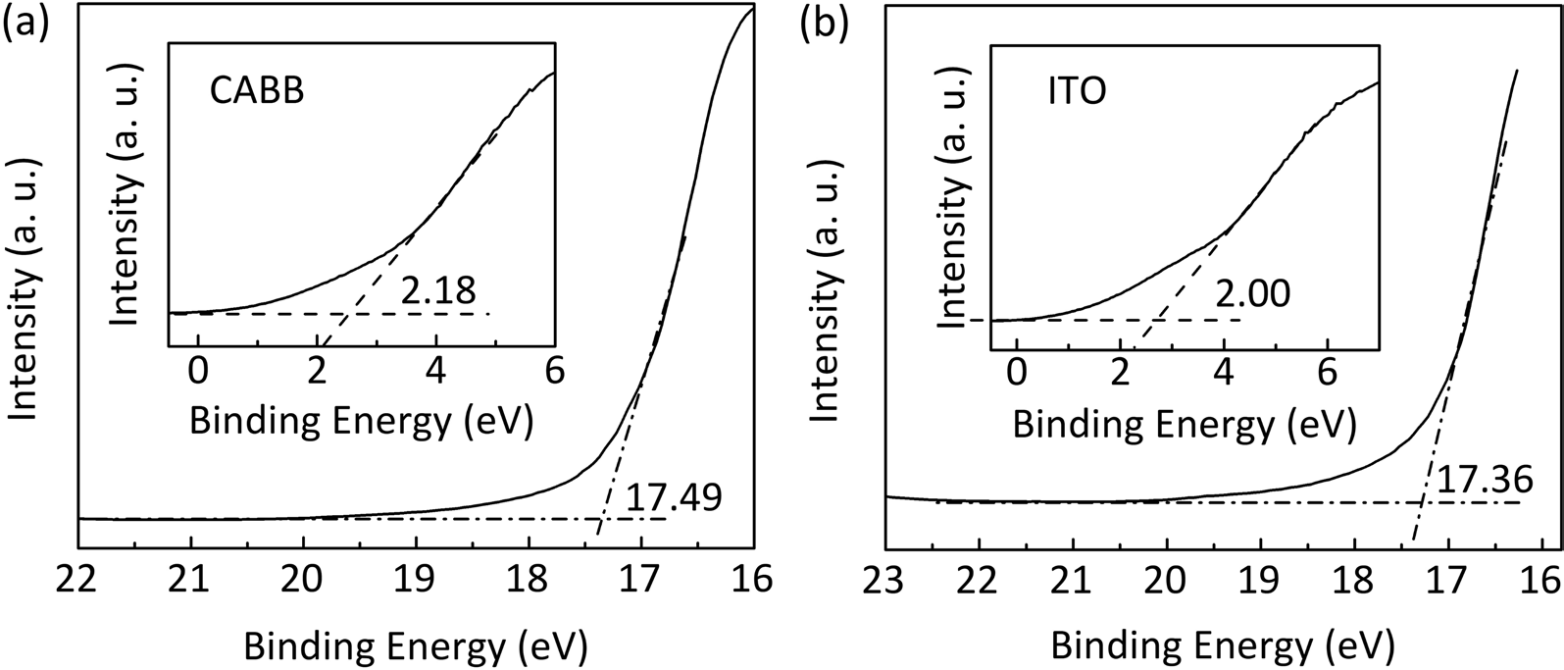
**a** UPS spectrum of the CABB film at cutoff region obtained with $h\nu =21.22$ eV. The inset depicts the Fermi edge of CABB. **b** Cutoff region of the ITO-coated glass. The inset displays the Fermi edge of the ITO layer

Moreover, the change of barrier located at the interface of electrodes and RS functional layers is also contributed to the RS behavior under light irradiation [39, 40]. Figure 7a, b shows the Fermi and cutoff regions of CABB and ITO in the UPS spectra, respectively. The work function of the CABB film and the ITO BE is calculated as 5.91 eV and 5.86 eV, respectively. Thus, the contact type between ITO and CABB is Ohmic. The Schottky-like barrier generates in the Pt/CABB interface due to the larger work function of Pt (6.42 eV) [41]. Figure 6d shows the initial schematic band diagram of the Pt/CABB junction in the dark condition. The energy band of the CABB layer bends upward and a Schottky-like barrier forms in the Pt/CABB interface due to the higher Fermi level (E$_\mathrm{f}$) of CABB. The barrier prevents electrons transferring across the Pt/CABB interface, and the Pt/CABB/ITO/glass device remains in HRS. When the positive $\textit{V}_\mathrm{SET}$ applied to the Pt TE, V$_\mathrm{Brs}$ gradually are filled by electrons injected from the ITO BE in the depletion region near the Pt/CABB interface. The width and height of the Schottky-like barrier is lowered [Fig. 6e]. In the meantime, the holes can inject from the Pt electrode across the Pt/CABB interface and fill the hole traps distributed near the valence band (VB) of CABB [39]. With the hole concentration increasing at the Pt/CABB interface, the E$_\mathrm{f}$ of CABB moves toward to the VB, causing the barrier further lowering. And thus, the Pt/CABB/ITO/glass device switches from HRS to LRS. When the device is exposed to light illumination with a wavelength of 445 nm ($\sim$2.79 eV), plenty of photoinduced electron–hole pairs generate in the CABB layer, and then, they are separated by the $E_\mathrm{ext}$. Photo-generated electrons jump into the conduction band (CB) and become free electrons because they obtain enough energy; photo-generated holes intensify the band bending and constantly facilitate electron transport across the junction with a thinner Schottky-like barrier, as shown in Fig. 6f. In the meantime, the hole traps can capture photo-generated holes, causing the E$_\mathrm{f}$ shifts toward VB and leaving a lower Schottky-like barrier. In addition, more holes inject from Pt electrode can also be trapped by the hole traps [39]. Hence, the height of barrier further decreases. Continuous illumination hinders the recombination of electron–hole pairs and further reduces the inhibiting effect of the barrier, so the devices remain in LRS. Under the negative $\textit{V}_\mathrm{RESET}$, V$_\mathrm{Brs}$ move toward the Pt/CABB interface, the barrier recovers its original height and width, and the device switches back to its original HRS.

## Conclusions

In summary, light-assisted tristate bipolar RS characteristics were observed in the Pt/CABB/ITO/glass memory devices. The maximum ON/OFF ratio was $\sim$500, and different resistance states could be steadily maintained over 1200 s. The RS behavior was principally attributed to the trap-controlled SCLC mechanism, and charge traps composed of V$_\mathrm{Brs}$ were considered to play a key role in forming the conductive paths. The modulation of the Pt/CABB Schottky-like barrier under light illumination was also responsible for the RS behavior of the devices in the carrier injection-trapped/detrapped process. These results illustrate the potential multilevel storage capacity of the lead-free double perovskites in the optoelectronic memory field.

## Supplementary Information

**Additional file 1**. Supplementary Information.

## Data Availability

All data generated and analyzed during this study are included in this article and the attached supporting information.

## Abbreviations

RRAM: Resistive switching random access memory
CABB: Cs_2_AgBiBr_6_
ITO: Indium tin oxide
RS: Resistive switching
TE: Top electrode
BE: Bottom electrode
ECM: Electrochemical metallization mechanism
VCM: Valence variation mechanism
TCM: Thermochemical mechanism
*I*–*V*: Current–voltage
SCLC: Space charge-limited current
LRS: Low resistance states
HRS: High resistance state
*V*_SET_: Set voltage
*V*_RESET_: Reset voltage
*V*_r_: Read voltage
V_Brs_: Bromine vacancies
CB: Conduction band
VB: Valence band
E_f_: Fermi level
*E*_ext_: External electric field
XRD: X-ray diffractometry
XPS: X-ray photoelectron spectroscopy
UPS: Ultraviolet photoelectron spectroscopy
SEM: Scanning electron microscopy

## References

[1] Waser R, Aono M (2007). Nanoionics-based resistive switching memories. Nat Mater.

[2] Zahoor F, Zulkifli TZA, Khanday FA (2020) Resistive random access memory (RRAM): an overview of materials, switching mechanism, performance, multilevel cell (mlc) storage, modeling, and applications. Nanoscale Res Lett 15(90) 10.1186/s11671-020-03299-9 PMC7176808 32323059

[3] Wang H, Meng FB, Cai YR, Zheng LY, Li YG, Liu YJ, Jiang YY, Wang XT, Chen XD (2013). Resistive switching phenomena in thin films: materials, devices, and applications. Adv Mater.

[4] Ding XX, Feng YL, Huang P, Liu LF, Kang JF (2019) Low-power resistive switching characteristic in HfO_2_/TiO_x_ Bi-layer resistive random-access memory. Nanoscale Res Lett 14(157) 10.1186/s11671-019-2956-4 PMC6509306 31073774

[5] Ye CQ, Peng Q, Li MZ, Luo J, Tang ZM, Pei J (2012). Multilevel conductance switching of memory device through photoelectric effect. J Am Chem Soc.

[6] Mao JY, Zhou L, Zhu XJ, Zhou Y, Han S (2019). Photonic memristor for future computing: a perspective. Adv Opt Mater.

[7] Prezioso M, Riminucci A, Graziosi P, Bergenti I, Rakshit R, Cecchini R, Vianelli A, Borgatti F, Haag N, Willis M, Drew AJ, Gillin WP, Dediu VA (2013). A single-device universal logic gate based on a magnetically enhanced memristor. Adv Mater.

[8] Liu SS, Jin C, Zheng DX, Pang X, Wang YC, Wang P, Zheng WC, Bai HL (2019). Ferroelectric field manipulated nonvolatile resistance switching in Al:ZnO/Pb(Mg_1/3_Nb_2/3_)_0.7_Ti_0.3_O_3_ heterostructures at room temperature. Phys Chem Chem Phys.

[9] Wang SK, Sun XW, Li GH, Jia CH, Li GQ, Zhang WF (2018) Study on the multi-level resistance-switching memory and memory-state-dependent photovoltage in Pt/Nd: SrTiO_3_ junctions. Nanoscale Res Lett 18 10.1186/s11671-018-2433-5 PMC5766446 29330736

[10] Russo P, Xiao M, Liang R, Zhou NY (2018). UV-induced multilevel current amplification memory effect in zinc oxide rods resistive switching devices. Adv Funct Mater.

[11] Choi J, Han JS, Jang HW (2018). Organic-inorganic hybrid halide perovskites for memories, transistors, and artificial synapses. Adv Mater.

[12] Gao S, Yi XL, Shang J, Liu G, Li RW (2019). Organic and hybrid resistive switching materials and devices. Chem Soc Rev.

[13] Kim HJ, Han JS, Kim SG, Kim SY, Jang HW (2019). Halide perovskites for resistive random-access memories. J Mater Chem C.

[14] Lv FZ, Ling K, Zhong TT, Liu FC, Liang XG, Zhu CM, Liu J, Kong WJ (2020). Multilevel resistive switching memory based on a CH_3_NH_3_PbI_3-x_Cl_3_ film with potassium chloride additives. Nanoscale Res Lett.

[15] Cheng XF, Qian WH, Wang J, Yu C, He J, Li H, Xu Q, Chen D, Li NJ, Lu JM (2019). Environmentally robust memristor enabled by lead-free double perovskite for high-performance information storage. Small.

[16] Ke WJ, Kanatzidis WG (2019). Prospects for low-toxicity lead-free perovskite solar cells. Nat Commun.

[17] Chu L, Ahmad W, Liu W, Yang J, Zhang R, Sun Y, Yang JP, Li XA (2019). Lead-free halide double perovskite materials: a new superstar toward green and stable optoelectronic applications. Nano-Micro Lett.

[18] Zhao XG, Yang DW, Ren JC, Sun YH, Xiao ZW, Zhang YZ (2018). Rational design of halide double perovskites for optoelectronic applications. Joule.

[19] Kamat PV, Bisquert J, Buriak J (2017). Lead-free perovskite solar cells. ACS Energy Lett.

[20] Lee DE, Kim SY, Jang HW (2020). Lead-free all inorganic halide perovskite quantum dots: review and outlook. J Eur Ceram Soc.

[21] Longo G, Mahesh S, Buizza LRV, Wright AD, Ramadan A, Abdi-Jalebi M, Nayak PK, Herz LM, Snaith HJ (2020). Understanding the performance limiting factors of Cs_2_AgBiBr_6_ double-perovskite solar cells. ACS Energy Lett.

[22] Greul E, Petrus ML, Bineka A, Docampob P, Being T (2017). Highly stable, phase pure Cs_2_AgBiBr_6_ double perovskite thin films for optoelectronic applications. J Mater Chem A.

[23] Pan WC, Wu HD, Luo JJ, Deng ZZ, Ge C, Chen C, Jiang XW, Yin WJ, Niu GD, Zhu L, Yin LX, Zhou Y, Xie QG, Ke XX, Sui ML, Tang J (2017). Cs_2_AgBiBr_6_ single-crystal x-ray detectors with a low detection limit. Nat Photonics.

[24] Lei LZ, Shi ZF, Li Y, Ma YY, Zhang F, Xu TT, Tian YT, Wu D, Lia WJ, Du GT (2018). High-efficiency and air-stable photodetectors based on lead-free double perovskite Cs_2_AgBiBr_6_ thin films. J Mater Chem C.

[25] Ning WH, Wang F, Wu B, Lu J, Yan ZB, Liu XJ, Tao YT, Liu JM, Huang W, Fahlman M, Hultman L, Sum TC, Gao F (2018). Long electron-hole diffusion length in high-quality lead-free double perovskite films. Adv Mater.

[26] Lv FZ, Zhong TT, Qin YF, Qin HJ, Wang WF, Liu FC, Kong WJ (2021). Resistive switching characteristics improved by visible-light irradiation in a Cs_2_AgBiBr_6_-based memory device. Nanomaterials.

[27] Ham S, Choi S, Cho H, Na SI, Wang G (2019). Photonic organolead halide perovskite artificial synapse capable of accelerated learning at low power inspired by dopamine-facilitated synaptic activity. Adv Funct Mater.

[28] Yuan YB, Huang JS (2016). Ion migration in organometal trihalide perovskite and its impact on photovoltaic efficiency and stability. Acc Chem Res.

[29] Guo F, Zhao MT, Xu K, Huan Y, Ge SP, Chen YM, Huang JH, Cui YM, Zhuang JC, Du Y, Feng HF, Hao WC (2019). Evidence for dynamic relaxation behavior of oxygen vacancy in Aurivillius Bi_2_MoO_6_ from dielectric spectroscopy during resistance switching. J Mater Chem C.

[30] Chen ZL, Yu Y, Jin LF, Li YF, Li QY, Li TT, Li J, Zhao HL, Zhang YT, Dai HT, Yao JQ (2020). Broadband photoelectric tunable quantum dot based resistive random access memory. J Mater Chem C.

[31] Lv FZ, Ling K, Wang WF, Chen P, Liu FC, Kong WJ, Zhu CM, Liu J, Long LZ (2019). Multilevel resistance switching behavior in PbTiO_3_/Nb:SrTiO_3_(100) heterostructure films grown by hydrothermal-epitaxy. J Alloy Compound.

[32] Chen J, Reed MA, Rawlett AM, Tour JM (1999). Large on-off ratios and negative differential resistance in a molecular electronic device. Science.

[33] Espejo GG, Padón DR, Luque R, Camacho L, Miguel G (2019). Mechanochemical synthesis of three double perovskite: Cs_2_AgBiBr_6_, (CH_3_NH_3_)_2_TlBiBr_6_ and Cs_2_AgSbBr_6_. Nanoscale.

[34] Zhu YY, Cheng PW, Shi J, Wang HJ, Liu Y, Xiong R, Ma HY, Ma HX (2019). Bromine vacancy redistribution and metallic-ion migration-induced air-stable resistive switching behavior in all-inorganic perovskite CsPbBr_3_ film-based memory device. Adv Electron Mater.

[35] Zhang GQ, Cai L, Zhang YF, Wei Y (2018). Bi^5+^, Bi^(3−x)+^, and oxygen vacancy induced BiOCl_x_I_1-x_ solid solution toward promoting visible-light driven photocatalytic activity. Chem Eur J.

[36] Gu C, Lee JS (2016). Flexible hybrid organic-inorganic perovskite memory. ACS Nano.

[37] Cai HZ, Lao MM, Xu J, Chen YK, Zhong CJ, Lu SR, Hao A, Chen RQ (2019). All-inorganic perovskite Cs_4_PbBr_6_ thin films in optoelectronic resistive switching memory devices with a logic application. Ceram Int.

[38] Azpiroz JM, Mosconi E, Bisquertcd J, Angelis FD (2015). Defect migration in methylammonium lead iodide and its role in perovskite solar cell operation. Energy Environ Sci.

[39] Zhou FC, Liu YC, Shen XP, Wang MY, Yuan F, Chai Y (2018). Low-voltage, optoelectronic CH_3_NH_3_Pbl_3−x_CI_x_ memory with integrated sensing and logic operations. Adv Funct Mater.

[40] Zheng PP, Sun B, Chen YZ, Elshekh H, Yu T, Mao SS, Zhu SH, Wang HY, Zhao Y, Yu Z (2019). Photo-induced negative differential resistance in a resistive switching memory device based on BiFeO_3_/ZnO heterojunctions. Appl Mater Today.

[41] Derry GN, Zhong ZJ (1989). Work function of Pt(111). Phys Rev B.

