# Correction to: Light-Activated Multilevel Resistive Switching Storage in Pt/Cs_2_AgBiBr_6_/ITO/Glass Devices

**DOI:** 10.1186/s11671-022-03659-7

**Published:** 2022-01-12

**Authors:** Tingting Zhong, Yongfu Qin, Fengzhen Lv, Haijun Qin, Xuedong Tian

**Affiliations:** School of Physical Science and Technology and Guangxi Key Laboratory of Nuclear Physics and Technology, Guangxi Normal University, Yucai Road, Guilin, 541000 China

## Correction to: Nanoscale Research Letters (2021) 16:178 https://doi.org/10.1186/s11671-021-03636-6

Following the publication of the original article [1], the authors flagged that the equal contribution symbol (†) had been (incorrectly) assigned to the third author in the author list. It has now been (correctly) assigned to the second author in the list, Yongfu Qin, in the original article.
