# Supplementary material for: Light-activated Multilevel Resistive Switching Storage in Pt/Cs2AgBiBr6/ITO/Glass Devices
Source: Nanoscale Res Lett. 2021 Dec 13;16:178. doi: 10.1186/s11671-021-03636-6 (PMC8669091; doi:10.1186/s11671-021-03636-6)
Supplement: Supplementary file 1 — Additional file 1. Supplementary Information. [file 11671_2021_3636_MOESM1_ESM.docx]

**Supporting Information**

**Light-activated multilevel resistive switching storage in Pt/Cs_2_AgBiBr_6_/ITO/Glass devices**

Tingting Zhong, Yongfu Qin^✝^, Fengzhen Lv^*^, Haijun Qin and Xuedong Tian

*School of Physical Science and Technology and Guangxi Key Laboratory of Nuclear Physics and Technology, Guangxi Normal University, Yucai Road, Guilin 541000, China*


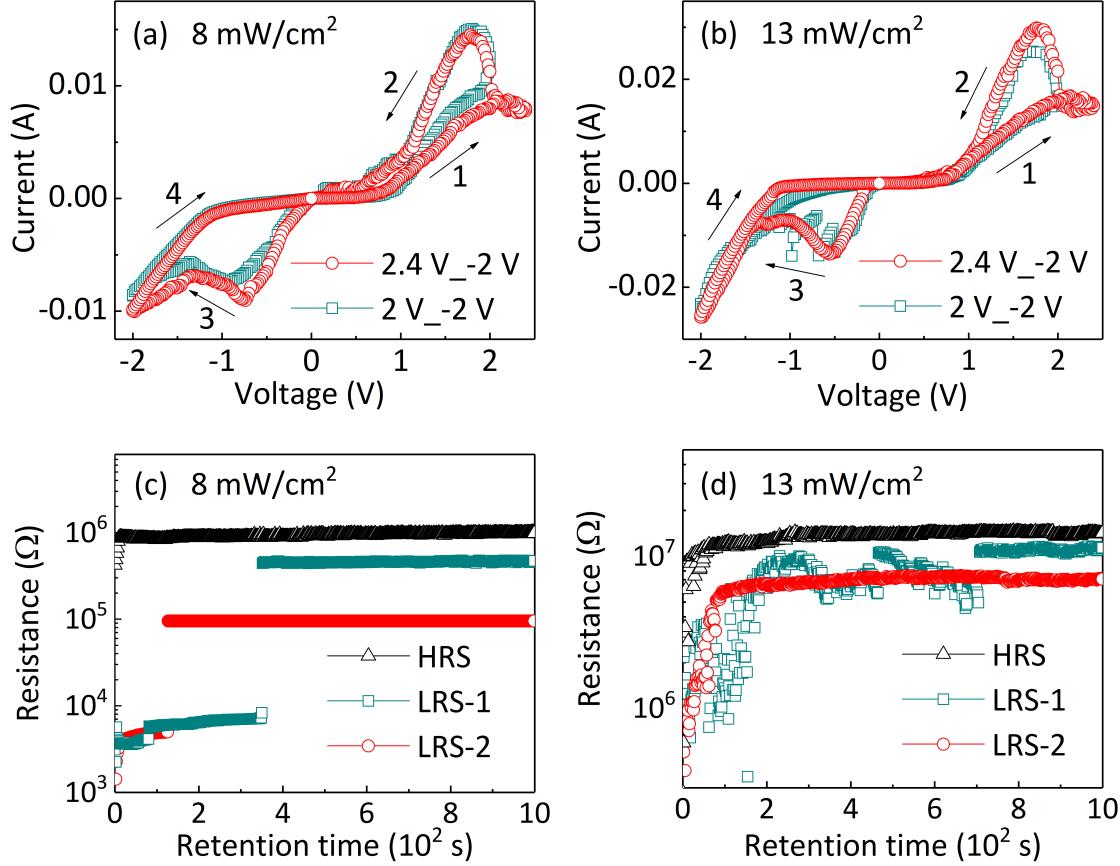


**Fig. S1** *I-V* curves of the Pt/CABB/ITO/glass device under illumination with (a) 8 mW/cm^2^ and (b) 13 mW/cm^2^. Resistances *vs.* time measured at -0.1 V after poling by +2.4 V, +2 V and -2 V under the light illumination of 445 nm with (c) 8 mW/cm^2^ and (d) 13 mW/cm^2^.
